# Retraction: Bismuth-based nanoparticles and nanocomposites: synthesis and applications

**DOI:** 10.1039/d5ra90024g

**Published:** 2025-03-11

**Authors:** Sujit Kumar, M. Premkumar, Jayant Giri, S. M. Mozammil Hasnain, Rustem Zairov, Jundao Wu, Zeai Huang

**Affiliations:** a Department of Electrical and Electronics Engineering, Dayananda Sagar College of Engineering Bengaluru Karnataka India sujit-eee@dayanandasagar.edu drpremkumar-eee@dayanandasagar.edu; b Department of Mechanical Engineering, Yeshwantrao Chavan College of Engineering Nagpur India jayantpgiri@gmail.com; c Division of Research and Development, Lovely Professional University Phagwara India; d Department of VLSI Microelectronics, Saveetha School of Engineering, Saveetha Institute of Medical and Technical Sciences (SIMATS), Saveetha University Chennai 602105 TN India; e Marwadi University Research Center, Department of Mechanical Engineering, Faculty of Engineering & Technology, Marwadi University Rajkot 360003 Gujarat India smmh.429@gmail.com; f Aleksander Butlerov Institute of Chemistry, Kazan Federal University 1/29 Lobachevskogo Str. Kazan 420008 Russian Federation rustem02@yandex.ru; g Arbuzov Institute of Organic and Physical Chemistry, FRC Kazan Scientific Center of RAS Arbuzov Str., 8 420088 Kazan Russian Federation; h State Key Laboratory of Oil and Gas Reservoir Geology and Exploitation, Southwest Petroleum University Chengdu 610500 China; i School of New Energy and Materials, Southwest Petroleum University Chengdu 610500 China zeai.huang@swpu.edu.cn; j Department of Electrical and Electronics Engineering, Visvesvaraya Technological University Belagavi Karnataka India

## Abstract

Retraction of ‘Bismuth-based nanoparticles and nanocomposites: synthesis and applications’ by Sujit Kumar *et al.*, *RSC Adv.*, 2024, **14**, 39523–39542, https://doi.org/10.1039/D4RA05637J.

The Royal Society of Chemistry, with the agreement of the authors, hereby wholly retracts this *RSC Advances* article.

Sujit Kumar has admitted that content from this review article originated from confidential information taken during the peer review process of an unpublished submission they were assigned to review from another publisher. None of the other co-authors were involved in this situation and bear no responsibility for this misconduct.

Signed: Sujit Kumar, Rustem Zairov, Jundao Wu, Zeai Huang, S. M. Mozammil Hasnain, Jayant Giri, M. Premkumar

Date: 4th March 2025

Retraction endorsed by Laura Fisher, Executive Editor, *RSC Advances*

